# Gold Nanoparticles-Based Colorimetric Immunoassay of Carcinoembryonic Antigen with Metal–Organic Framework to Load Quinones for Catalytic Oxidation of Cysteine

**DOI:** 10.3390/s24206701

**Published:** 2024-10-18

**Authors:** Zhao-Jiang Yu, Ting-Ting Yang, Gang Liu, De-Hua Deng, Lin Liu

**Affiliations:** College of Chemistry and Chemical Engineering, Anyang Normal University, Anyang 455000, China; yzj86jiang@aynu.edu.cn (Z.-J.Y.); 15670202606@163.com (T.-T.Y.); liugang08215@163.com (G.L.); ddh@aynu.edu.cn (D.-H.D.)

**Keywords:** colorimetric immunoassays, gold nanoparticles, redox cycling, metal–organic frameworks, quinone, cysteine

## Abstract

This work reported gold nanoparticles (AuNPs)-based colorimetric immunoassay with the Cu-based metal–organic framework (MOF) to load pyrroloquinoline quinone (PQQ) for the catalytic oxidation of cysteine. In this method, both Cu^2+^ and PQQ in the MOF could promote the oxidation of inducer cysteine by redox cycling, thus limiting the cysteine-induced aggregation of AuNPs and achieving dual signal amplification. Specifically, the recombinant carcinoembryonic antigen (CEA) targets were anchored on the MOF through the metal coordination interactions between the hexahistidine (His_6_) tag in CEA and the unsaturated Cu^2+^ sites in MOF. The CEA/PQQ-loaded MOF could be captured by the antibody-coated ELISA plate to catalyze the oxidation of cysteine. However, once the target CEA in the samples bound to the antibody immobilized on the plate surface, the attachment of CEA/PQQ-loaded MOF would be limited. Cysteine remaining in the solution would trigger the aggregation of AuNPs and cause a color change from red to blue. The target concentration was positively related to the aggregation and color change of AuNPs. The signal-on competitive plasmonic immunoassay exhibited a low detection limit with a linear range of 0.01–1 ng/mL. Note that most of the proteins in commercial ELISA kits are recombinant with a His_6_ tag in the N- or C-terminal, so the work could provide a sensitive plasmonic platform for the detection of biomarkers.

## 1. Introduction

Immunoassays have witnessed huge progress in diverse applications such as disease diagnosis, food safety monitoring, and environmental protection due to the highly specific antigen–antibody interactions [1]. A labeling element is usually required to convert the specific recognition event into a certain measurable signal for the transduction readout. According to the type of signal output, immunoassays can be classified into fluorescence, colorimetry, surface plasmon resonance, surface-enhanced Raman scattering, electrochemistry, electrochemiluminescence, and photoelectrochemistry [2,3]. Among them, colorimetric immunoassay is one of the most commonly used and favored methods due to the distinct advantages of straightforward readout, simple operation, and minimal requirement for instrumentation [4,5]. It has been utilized for the detection of different targets, such as small molecules, proteins, enzymes, exosomes, and whole cells [6]. In a classic colorimetric immunoassay, enzymes (e.g., horseradish peroxidase and alkaline phosphatase) conjugated with the detection antibodies were employed to produce a detectable signal via the enzymatic conversion of chromogenic substrates into easily detectable products, including 2,2′-azino-bis-(3-ethylbenzthiazoline-6-sulfonic acid) (ABTS), 3,3′,5,5′-tetramethylbenzidine (TMB), and *o*-phenylenediamine (OPD) [7,8,9,10,11]. However, several limiting factors may result in poor signal-to-noise ratio and low sensitivity, such as the low enzyme/antibody ratio, low extinction coefficient of small molecular substrates, and high cost and short shelf life of enzymes.

Aiming to enhance the assay sensitivity, nanomaterials with excellent physical and chemical properties have been combined with colorimetric immunoassays for signal amplification. For example, nanomaterials with a large surface area, such as magnetic nanobeads and mesoporous silica nanoparticles, can serve as nanocarriers to load multiple enzymes, antibodies, and signal reporters [6,12,13]. Nanoparticles composed of special metal ions or organic elements can act as sacrificial labels to release massive institute components and further trigger the chromogenic reactions [14,15,16]. Typically, Bao’s group reported a signal amplification strategy for immunoglobulin G detection based on the acid-induced dissolution of iron oxide nanoparticles and Fe^3+^-catalyzed chromogenic Fenton reaction [17]. Additionally, a large number of nanomaterials with enzyme-like activity, known as nanozymes, have been used as enzyme-mimicking labels to catalyze chromogenic reactions for signal amplification [7,18,19,20]. Compared with the natural enzymes, nanozymes possess excellent advantages, including being less vulnerable to denaturation and protease digestion, low cost, and ease of mass production. For instance, Xia et al. used peroxidase-like Pd-Ir core-shell nanocubes as the labels to develop a colorimetric immunoassay method for prostate-specific antigen (PSA) detection, whose detection limit was approximately 100-fold lower than that of the conventional HRP-based immunoassay [21]. However, most of these methods still require the use of small chromogenic substrates, and their low extinction coefficient will lead to weak color change for the detection of ultralow concentration of targets.

Because of the unique localized surface plasmon resonance (LSPR), noble metal nanomaterials exhibit 1000 times higher extinction coefficients compared with organic dyes, leading to the higher detection sensitivity [22,23,24,25]. Typically, plasmonic gold and silver nanoparticles have been popularly used as chromogenic indicators for colorimetric immunoassays [26,27]. Various factors, including the size, shape, composition, and distance between the nearby nanoparticles, can alter the intensity and frequency of the LSPR band, eventually inducing an obvious change in the absorbance intensity and solution color. Among them, gold nanoparticles (AuNPs) aggregation-based colorimetric methods have been reported for the detection of small molecules, ions, and proteins [26]. Cysteine (Cys) can bind to soft gold or silver through the metal–thiol interaction and induce the aggregation of AuNPs via intermolecular hydrogen bonds or electrostatic interactions [28,29,30,31]. The Cys-mediated AuNPs aggregation has been coupled with other signal amplification strategies by means of the enzymatical or non-enzymatical modulation of change in Cys concentration [32,33,34,35,36,37]. For example, Liu et al. reported AuNP-based colorimetric immunoassay by integrating alkaline phosphatase catalysis, Fe^2+^-catalyzed Cys oxidation, and Cys-stimulated AuNPs aggregation [36]. Abbas et al. developed a colorimetric immunoassay for pathogen detection based on the controlled release of Cys from liposome to trigger AuNP aggregation [37]. Therefore, it would be interesting to integrate more powerful cascade amplification strategies into AuNP-based colorimetric immunoassays.

Metal–organic frameworks (MOFs), a typical class of porous crystalline materials, are composed of organic ligands and metal ions or clusters. The diversity of organic ligands and redox-active metal ions (e.g., Fe^2+^, Cu^2+^, Co^2+^, Ce^3+^, and Mn^2+^) make MOFs promising enzyme mimics for colorimetric bioassays [38,39,40,41]. In the presence of H_2_O_2_ or dissolved O_2_, MOFs can oxidize different substances, including chromogenic substrates, ascorbic acid, and Cys [40,42]. Additionally, MOFs exhibit a large surface area-to-volume ratio, ease of bioconjugation, and flexible structures, which are favorable for them serving as nanocarriers to accumulate dyes, redox-active molecules, and biorecognition elements [43,44,45,46]. It has been suggested that small organic molecules such as quinone and fluorescein can catalyze the oxidation of different reagents, including Cys, tris(2-carboxyethyl)phosphine (TCEP), and TMB through redox cycling reaction or enzyme-like catalysis [47,48,49,50,51,52,53]. Thus, it is promising to integrate enzyme-mimicking MOFs with small molecular catalysts for more effective catalysis.

Carcinoembryonic antigen (CEA) is a type of non-specific and broad-spectrum cancer biomarker commonly utilized for early cancer diagnosis and treatment efficacy evaluation tumor [54]. In this work, AuNP-based enzyme-free colorimetric immunoassays were constructed for the detection of CEA with multiple signal amplification, in which Cu-MOF was used as the nanocarrier to load catalytically active pyrroloquinoline quinone (PQQ) and recombinant CEA. The signal tags could be specifically captured by the antibody-covered plate to promote the oxidation of Cys under the simultaneous catalysis of Cu-MOF and PQQ, resulting in effective inhibition of Cys-triggered AuNPs aggregation. The AuNP-based enzyme-free immunoassays exhibited the advantages of high sensitivity, naked-eye readout, and simplicity. The strategy also offers great potential for the design of novel signal tags for bioassays.

## 2. Materials and Methods

### 2.1. Reagents and Materials

Cys, PQQ, gold(III) chloride trihydrate, trisodium citrate dehydrate, Cu(NO_3_)_2_ hydrate, N,N-dimethylformamide (DMF), 1,3,5-benzenetricarboxylic acid (BTC), 5,5-dithiobis-(2-nitrobenzoic acid) (DTNB), and polyvinyl pyrrolidone (PVP) were obtained from Aladdin Chemical Technology Co., Ltd. (Shanghai, China). Bovine serum albumin (BSA), thrombin, and sodium phosphate were purchased from Sigma-Aldrich (Shanghai, China). Prostate-specific antigen (PSA), CEA, and ELISA kits were provided by Linc-Bio Science Co. Ltd. (Shanghai, China). AuNPs with an average size of 13 nm were synthesized with a citrate reduction method. Other chemical reagents were of analytical grade and used without additional purification. Ultrapure water used in experiments was prepared by a Milli-Q purification system.

### 2.2. Synthesis and Characterization of Cu-BTC MOF

Cu-BTC MOF was prepared according to the previously reported method [55]. Specifically, Cu(NO_3_)_2_ hydrate (0.875 g) and PVP (0.3 g) were dissolved in 25 mL DMF and BTC (0.42 g) was dissolved in 25 mL DMF. The two solutions were then mixed and stirred for 10 min. After that, the mixture was transferred into a Teflon-lined autoclave and heated at 100 °C for 16 h. The resulting resultants were centrifugated at 5000 rpm for 10 min and washed with DMF and ethanol several times. The solid product was dried at 60 °C for 12 h under vacuum. The morphology of Cu-BTC was imaged on a Hitachi SU8010 scanning electron microscopy (SEM, Tokyo, Japan), and its average size was determined by a Malvern Zetasizer Nano ZS model ZEN 3600 (Worcestershire, UK).

### 2.3. Determination of Surface Area and Pore Volume of MOF

The surface area and pore volume of MOF were determined by low-temperature nitrogen adsorption–desorption analysis (BSD-PS2, BSD instrument Technology (Beijing, China) Co., Ltd.). In brief, samples were degassed under vacuum at 120 °C for 2 h prior to analysis. Nitrogen sorption experiments were conducted at −196 °C. The surface area was determined by multipoint Brunauer–Emmett–Teller (BET) adsorption characteristics at a relative pressure (p/p_0_, equilibrium pressure of nitrogen at the sample surface/saturation pressure of nitrogen) between 0.04 and 0.32. The pore volume was obtained at a relative pressure of p/p_0_ > 0.35. The overall pore volume was reported based on the BJH adsorption cumulative volume of pores between 1.7 and 195.6 nm width.

### 2.4. Preparation of CEA@Cu-BTC@PQQ

The prepared Cu-BTC MOF was dispersed in phosphate buffer (10 mM, pH 7.4) at the concentration of 1 mg/mL. Then, 1 mL of 10 mM PQQ dissolved in 0.1 M NaOH was added to 100 mL of Cu-BTC suspension with a final concentration of about 0.1 mM. The mixture was gently stirred for 2 h and the product was collected by centrifugation at 8000 rpm for 5 min and washed with the buffer three times to remove free PQQ. The amount of PQQ loaded onto the MOF was determined to be 83 nmol/mg by measuring the level of free, unentrapped PQQ in the solution with differential pulse voltammetry. The resulting Cu-BTC@PQQ was characterized by Nicolet iS10 Fourier-transform infrared (FTIR) spectrophotometer (Thermo Scientific, Inc., Waltham, MA, USA), Nicolet iS50 Ultima III X-ray power diffractometer (XRD), and Thermo ESCALAB 250Xi X-ray photoelectron spectroscopy (XPS).

For the preparation of CEA@Cu-BTC@PQQ, 0.5 mL of Cu-BTC@PQQ (0.1 mg/mL) was mixed with 0.5 mL of recombinant CEA (0.1 mg/mL) and shaken slightly at room temperature for 30 min. After that, the free CEA was removed by centrifugation at 8000 rpm for 5 min and washing the solid three times. The recombinant CEA was anchored on the MOF through the coordination interactions between the His_6_ tag in CEA and the unsaturated metal sites in Cu-BTC MOF [56]. The resulting CEA@Cu-BTC@PQQ was dispersed in 10 mL phosphate buffer for use.

### 2.5. Monitoring of Cys Oxidation

The catalytic oxidation of Cys was monitored by using AuNPs and DTNB as the indicators, respectively. In the AuNP-based assays, 100 μL of 50 μM Cys was incubated with 100 μL of 0.2 μM PQQ, 2 μg/mL Cu-BTC, or 2 μg/mL Cu-BTC@PQQ for 60 min. Then, 100 μL of the prepared AuNPs suspension was added to the mixtures. After incubation for 10 min, the color change was observed by naked-eye and the absorption spectra were recorded on Agilent Cary 60 UV-Vis spectrophotometer (Suzhou, China). In the DTNB-based assays, 100 μL of 500 μM Cys was incubated with 100 μL of 2 μM PQQ, 20 μg/mL Cu-BTC, or 20 μg/mL Cu-BTC@PQQ for 60 min. Then, 100 μL of 1 mM DTNB solution at a concentration of 1 mM was added to the mixtures. After reaction for 10 min, the absorption spectra were collected in the range of 350~500 nm.

### 2.6. Electrochemical Measurement

The voltammetric responses of PQQ at different pH values were recorded on a CHI 660 electrochemical workstation using a homemade plastic electrochemical cell. Prior to each experiment, the glassy carbon electrode (3 mm in diameter) was successively polished with 1 and 0.3 μm diamond pastes and then sonicated in deionized water. A phosphate buffer (10 mM, pH 5.5 or 8.2) containing 50 mM Na_2_SO_4_ was used as the electrolyte solution.

### 2.7. Colorimetric Immunoassays of CEA

The colorimetric immunoassays of CEA were performed by using a conventional ELISA kit (Linc-Bio Science Co. Ltd., Shanghai, China). A total of 100 μL of CEA standard solution at a given concentration was spiked onto the 96-well microplate coated with the CEA capture antibody. After incubation for 30 min, the plate was washed three times. Then, 100 μL of the prepared CEA@Cu-BTC@PQQ suspension was added for incubation of 30 min. After washing the plate three times, 100 μL of 20 μM Cys solution was added and reacted with the captured Cu-BTC@PQQ for 60 min. After that, 50 μL of AuNPs suspension was supplemented to the solution for ten-minute incubation. The photograph was taken and the absorption spectrum was recorded on the Cary 60 UV-Vis spectrophotometer.

## 3. Results and Discussion

### 3.1. Detection Principle

**Scheme 1:**
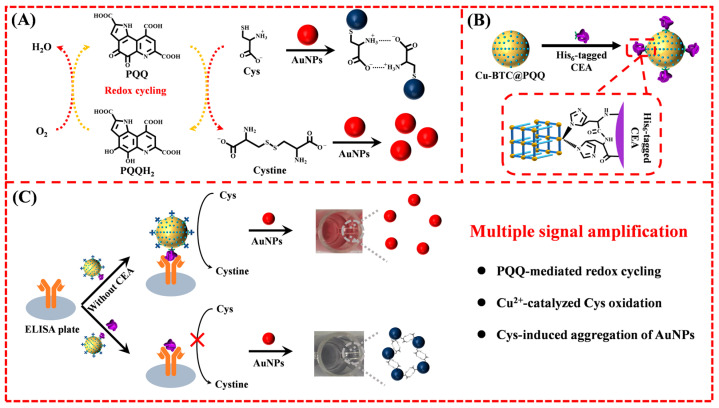
Schematic illustration of PQQ-promoted catalytic oxidation of Cys (**A**), modification of Cu-BTC@PQQ with recombinant CEA (**B**), and AuNP-based colorimetric immunoassays (**C**).

Small molecules and metal ions could be encapsulated inside the inner cavity or absorbed on the surface of nanocarriers through non-covalent or encapsulation interactions [2,12,40]. They would be released under a certain stimulation to directly produce a colorimetric signal or indirectly trigger the color change of additional substances. Cysteine could induce the rapid aggregation of AuNPs, thus allowing for the development of colorimetric immunoassays [36,37]. Redox-active metal ions and metallic nanoparticles could catalyze the oxidation of reducing reagents including cysteine. In addition, quinones can promote the oxidation of cysteine based on redox cycling reactions (Scheme 1A) [49]. Inspired by these facts, we developed a colorimetric immunoassay method by using PQQ-loaded Cu-BTC MOF to catalyze the oxidation of Cys and prevent the Cys-triggered aggregation of AuNPs. Immunosensors have been mainly designed in a sandwich or competitive format. Generally, the signal-on method exhibits higher sensitivity than the signal-off format. For this view, the competitive colorimetric immunoassay was designed in a signal-on format and CEA was determined as a model analyte. As shown in Scheme 1B, recombinant CEA was anchored on the PQQ-loaded Cu-BTC MOF through the metal ion-His_6_ interactions [56]. In the absence of target CEA, the resulting CEA@Cu-BTC@PQQ could be captured by the antibody-coated ELISA plate (Scheme 1C), thus catalyzing the oxidation of Cys by O_2_. In this case, AuNPs were monodispersed and the solution showed red color. However, the attachment of target CEA onto the surface of antibody-coated ELISA plate would prevent the anchoring of CEA@Cu-BTC@PQQ, thereby limiting the catalytic oxidation of Cys. Consequently, the Cys-triggered aggregation of AuNPs was prevented due to the presence of target analyte. In this work, both Cu-BTC and PQQ could catalyze the oxidation of Cys, thus achieving dual signal amplification. Note that most of the commercial proteins in ELISA kits are recombinant with a His_6_ tag at the N or C-terminal; the proposed strategy could be used for the design of different immunosensors by immobilizing the recombinant antigens on the surface of nanolabels through metal coordination interactions.

### 3.2. Characterization of Cu-BTC@PQQ

**Figure 1:**
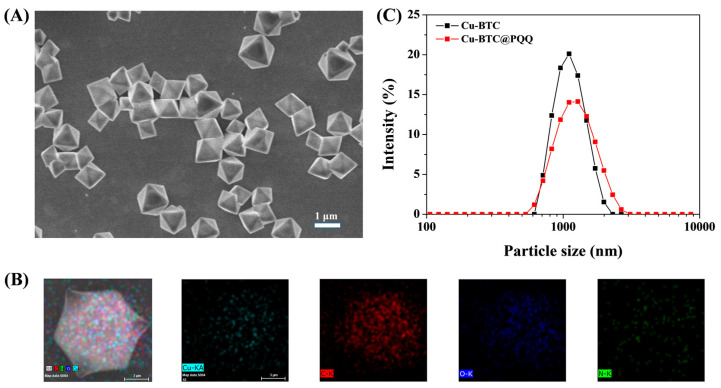
SEM (**A**) and EDS elemental mapping (**B**) pictures of Cu-BTC MOF. (**C**) DLS data of Cu-BTC and Cu-BTC@PQQ.

**Figure 2:**
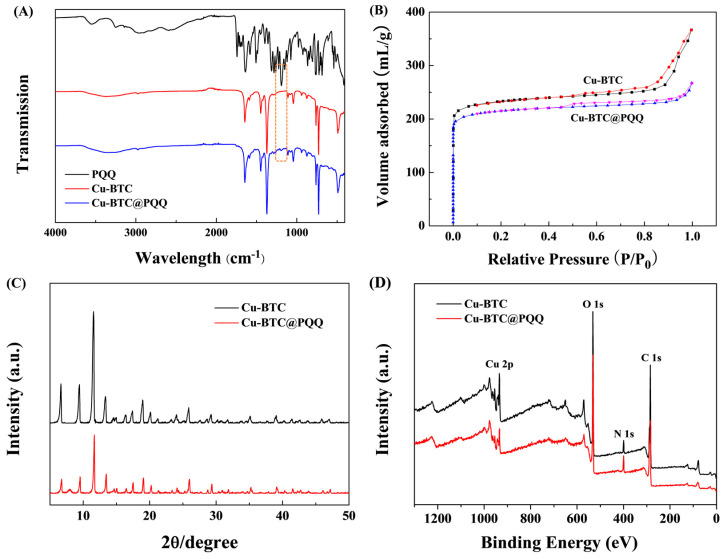
(**A**) FTIR spectra of PQQ (black curve), Cu-BTC (red curve), and Cu-BTC@PQQ (blue curve). (**B**) BET curves, (**C**) XRD patterns, and (**D**) XPS spectra of Cu-BTC and Cu-BTC@PQQ.

The morphology of Cu-BTC was first characterized by SEM. As shown in Figure 1A, the MOF with a regular octahedral geometry was relatively uniformly dispersed. The EDS elemental mapping images suggested that they were composed of Cu, C, O, and N elements that are well distributed in the particles (Figure 1B). The DLS results demonstrated that the average diameter of Cu-BTC MOF was 1126 nm (Figure 1C), and the value was intensified to 1323 nm after the loading of PQQ. The size measured by DLS was larger than that found by SEM, which is understandable since the DLS data were derived from the hydration radius but not the true size of particles. To further prove that PQQ was loaded in the MOF, FTIR spectra of Cu-BTC MOF before and after incubation with PQQ were collected. As shown in Figure 2A, the small characterized absorption peaks located at the range of 1320~1120 cm^−1^ for Cu-BTC@PQQ could be attributed to the loading of PQQ. In addition, the change in the surface area and pore volume of Cu-BTC MOF after the loading of PQQ was studied by BET analysis (Figure 2B). The surface area decreased from 1047 m^2^/g to 960 m^2^/g and the pore volume changed from 0.5544 mL/g to 0.3976 mL/g after the loading of PQQ. These results suggested that PQQ molecules were successfully loaded into the Cu-BTC MOF. Furthermore, XRD and XPS were used to characterize the crystal structure and constituent element of Cu-BTC before and after the entrapping of PQQ. As shown in Figure 2C, the characteristic XRD peaks are consistent with those in the previous report [55], indicating that the prepared Cu-BTC MOF has good morphology, good purity, and high crystallinity. No significant change in the characteristic of XRD pattern was observed after the loading of PQQ, suggesting that the entrapping of PQQ did not destroy the morphology and structure of Cu-BTC. In addition, the XPS spectrum confirmed that both Cu-BTC and Cu-BTC@PQQ were composed of Cu, C, O, and N elements. It is worth noting that both EDS and XPS results indicated the existence of N elements in the MOF although no N atom is included in the ligand of BTC. The result is understandable since PVP was used as the dispersing agent during the preparation of Cu-BTC MOF. Thus, the N element should be attributed to PVP adsorbed onto the MOF.

### 3.3. Catalytic Oxidation of Cys

**Figure 3:**
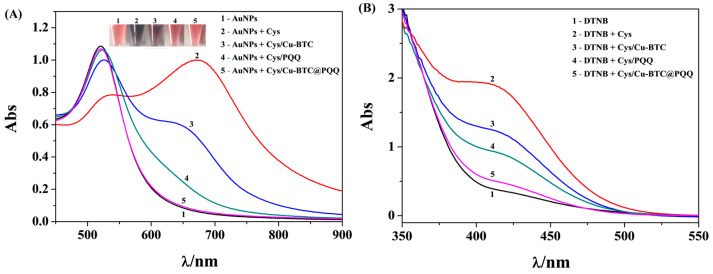
UV-Vis absorption spectra of AuNPs (**A**) and DTNB (**B**) in the absence and presence of Cys, Cys/Cu-BTC, Cys/PQQ, and Cys/Cu-BTC@PQQ. The inset in panel A shows the corresponding photographs of AuNPs in different systems.

The previous investigations have indicated that PQQ could rapidly accelerate the oxidation of Cys in the presence of O_2_ [57], and Cys but not its oxidized format (cystine) could induce the aggregate of AuNPs with the color change from red to blue [36,37]. Herein, we attempted to construct a sensitive AuNP-based plasmonic ELISA platform based on the Cu-based MOF and PQQ-mediated oxidation of Cys and the aggregation of AuNPs. To achieve the goal, we first investigated the Cys-triggered color and absorption changes of AuNPs suspension in the absence and presence of PQQ and Cu-BTC. As shown in Figure 3A, the dispersed AuNPs with red color exhibited an absorption peak at 520 nm (tube/curve 1). After the addition of Cys, the solution color became purple or even blue and the absorption peaked at 650 nm (tube/curve 2), which is attributed to the Cys-induced aggregation of AuNPs. The shift was dependent on the concentration of Cys in the range of 1~20 μM, which is consistent with that of previous reports [28,30,31]. When Cys was incubated with Cu-BTC (tube/curve 3), PQQ (tube/curve 4), and Cu-BTC@PQQ (tube/curve 5) for a given time, the color and absorption changes were inhibited to a certain extent. Ellman’s method is a classical and widely used strategy for the detection of thiols [58]. To prove that the aggregation of AuNPs was mediated by Cys, Ellman’s method was used to monitor the catalytic oxidation of Cys by Cu-BTC and PQQ with DTNB as the indicator. As shown in Figure 3B, DTNB could be reduced into 5-thio-(2-nitrobenzoic acid) (TNB) by Cys, thus causing the presence of an absorption peak at 412 nm (curves 1 and 2). Incubation of Cys with Cu-BTC, PQQ, and Cu-BTC@PQQ (curves 3, 4 and 5) limited the increase in the absorption intensity at 412 nm. These results indicated that both Cu-BTC and PQQ could catalyze the oxidation of Cys and prevented the Cys-triggered aggregation of AuNPs.

### 3.4. Optimization of Catalytic Conditions

**Figure 4:**
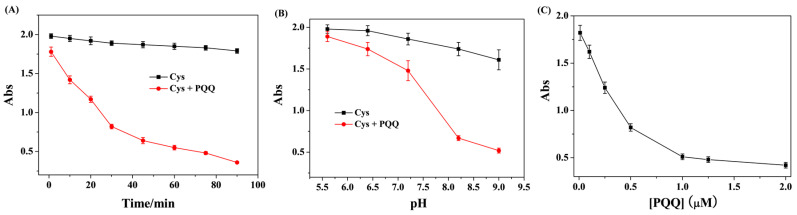
Effect of incubation time (**A**) and pH (**B**) on the oxidation of Cys in the absence (black dotted curve) and presence (red dotted curve) of PQQ. (**C**) Dependence of absorption intensity on PQQ concentration.

The rate for catalytic oxidation of Cys by PQQ was dependent upon reaction time and solution pH value. To optimize the reaction conditions, the effect of incubation time and pH value on the PQQ-catalytic oxidation of Cys was examined by Ellman’s method. As shown in Figure 4A, the absorption intensity was higher and showed slight dependence on the incubation time, indicating that Cys was stable in the absence of PQQ. However, the absorption intensity decreased significantly with the increase of incubation time between Cys and PQQ, and began to level off beyond 45 min. After incubation for 90 min, the value reached the background from DTNB. In the following assays, 60 min was selected as the incubation time for the catalytic oxidation of Cys in order to save detection time and facilitate time-recording. We also examined the effect of pH value on the PQQ-catalytic oxidation of Cys. As shown in Figure 4B, the absorption intensity decreased significantly when Cys was incubated with PQQ at alkaline pH value. Considering that Gys may suffer from automatic oxidation in air, especially in an alkaline solution, in this work, a compromising pH of 8.2 was used for the catalytic oxidation of Cys. In addition, we investigated the effect of PQQ concentration on the oxidation of Cys by Ellman’s method and found that the absorption intensity decreased with the increase of PQQ concentration (Figure 4C), further indicating that the catalytic oxidation of Cys is initiated by PQQ-dependent redox cycling.

**Figure 5:**
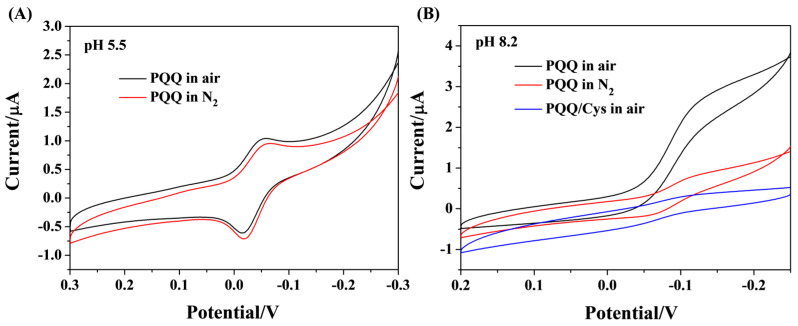
CVs of PQQ at pH 5.5 (**A**) and 8.2 (**B**) in air and N_2_-saturated solution. The blue curve in panel B shows that of PQQ/Cys in air-saturated solution. The concentrations of PQQ and Cys were 100 and 500 μM, respectively.

To explore the mechanism, we examined the voltammetric responses of PQQ at acidic and alkaline pH values. As shown in Figure 5A, the voltammogram of PQQ at pH 5.5 exhibited a couple of reversible redox waves in either air (black curve) or N_2_ (red curve) saturated solution, which is attributed to the redox of PQQ/PQQH_2_ with *E*_pa_ = −18 mV and *E*_pc_ = −60 mV. At pH 8.2 and higher (Figure 5B), the reduction peak at −142 mV was enhanced at the expense of the oxidation wave in the presence of O_2_ (black curve). When the solution was saturated by N_2_, the redox process became quasi-reversible (red curve). The results indicated that the sigmoidal voltammetric wave was indicative of a catalytic process in which the electrogenerated PQQH_2_ was rapidly re-oxidized by dissolved O_2_. After the addition of Cys, the reduction peak was reduced and the oxidation peak was intensified, indicating that PQQ could be reduced into PQQH_2_ by Cys although PQQH_2_ would be re-oxidized into PQQ by O_2_. The result demonstrated that the oxidation of Cys by O_2_ could be initiated by PQQ through the redox cycling reaction.

### 3.5. Feasibility for CEA Detection

**Figure 6:**
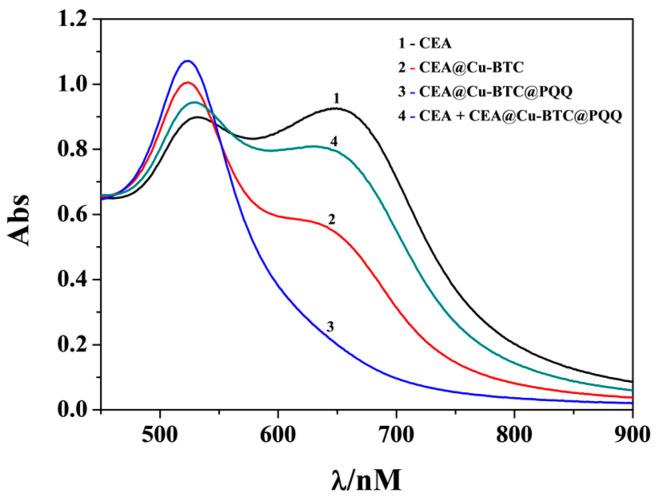
UV-Vis absorption spectra and photographs of the antibody-coated plates after treatment by CEA, CEA@Cu-BTC, CEA@Cu-BTC@PQQ, and CEA + CEA@Cu-BTC@PQQ, and then incubation with 20 μM Cys for 1 h before the addition of AuNPs. The concentration of CEA was 5 ng/mL.

To investigate the feasibility of the method for CEA detection, the antibody-coated plates were treated with different substances and then Cys and AuNPs were successively added to the pores. As depicted in Figure 6, when the plate was treated with CEA, the solution color became blue and the absorption peaked at 650 nm (tube/curve 1), which is attributed to the Cys-triggered aggregation of AuNPs. When the plate was treated with CEA@Cu-BTC or CEA@Cu-BTC@PQQ, the solution color became purple or red and the absorption intensity at 650 nm decreased or disappeared (tubes/curves 2 and 3), indicating that the captured Cu-BTC and Cu-BTC@PQQ could catalyze the oxidation of Cys and thus prevent the aggregation of AuNPs to a certain extent. Interestingly, when the plate was incubated with CEA before the capture of CEA@Cu-BTC@PQQ, the solution color became blue and the absorption peaked at 650 nm. This indicated that the attachment of CEA could limit the capture of CEA@Cu-BTC@PQQ, thus allowing for the Cys-triggered aggregation of AuNPs.

### 3.6. AuNP-Based Plasmonic ELISA for CEA Detection

**Figure 7:**
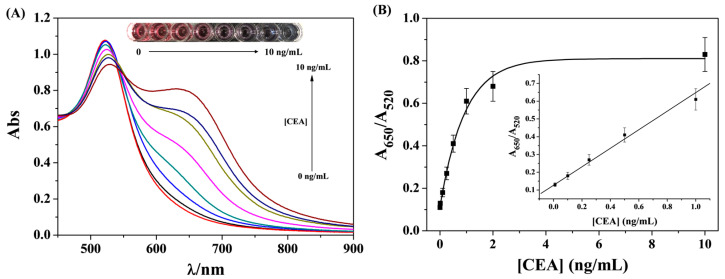
(**A**) UV-Vis absorption spectra and photographs for the assays of different concentrations of CEA (0, 0.01, 0.1, 0.25, 0.5, 1, 2, and 10 ng/mL). (**B**) Dependence of A_650_/A_520_ on CEA concentration. The inset shows the linear portion of the fitting curve.

The analytical performances of the AuNP-based plasmonic ELISA were examined by determining different concentrations of CEA. As depicted in Figure 7A, the absorption intensity at 650 nm was enhanced at the expense of that at 520 nm. The A_650_/A_520_ value was intensified proportionally with increasing CEA concentration (Figure 7A). The linear range covered 0.01–1 ng/mL of CEA with an equation of Y = 0.13 + 0.52 [CEA] (ng/mL). The lowest detectable concentration was as low as 0.01 ng/mL, which was low enough to meet the requirement of CEA detection [59]. The proposed plasmonic immunoassay was comparable to or better than most of the previously reported methods based on the enzymes or nanozymes-catalytic chromogenic reactions [60,61,62,63,64,65,66,67,68,69,70] and the release of dyes or metal ions to produce colorful metal complexes [71,72,73,74,75,76,77] (Table 1). The high sensitivity can be contributed to the dual signal amplification by Cu-BTC and PQQ-based catalysis reactions.

### 3.7. Selectivity

**Figure 8:**
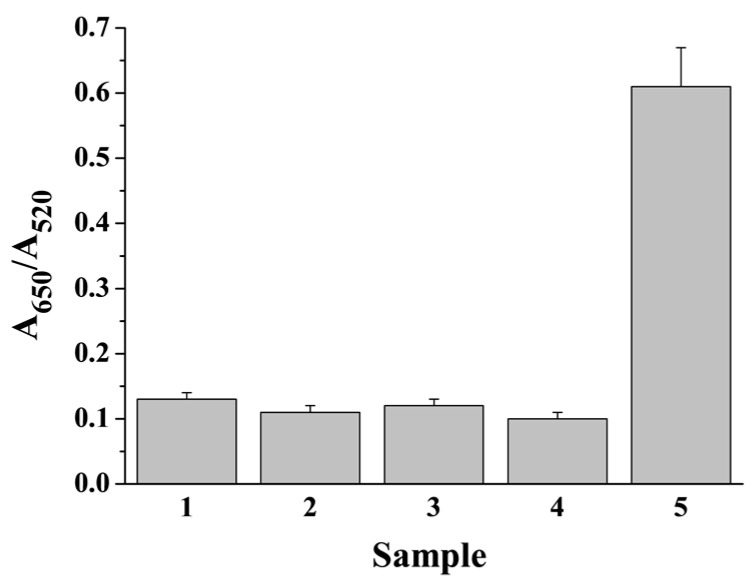
A_650_/A_520_ for the assays of 10 ng/mL BSA (bar 1), 10 ng/mL PSA (bar 2), 10 ng/mL thrombin (bar 3), 10% FBS (bar 4), and 1 ng/mL CEA (bar 5).

To investigate the selectivity of the method, we challenged the plasmonic immunoassays with three non-target proteins (BSA, PSA, and thrombin) and fetal bovine serum (FBS) (Figure 8). The A_650_/A_520_ values for the assays of the non-target proteins (bars 1~3) and FBS (bar 4) were close to the blank level and significantly lower than that of CEA target (bar 5), indicating that the plasmonic immunoassay platform could determine CEA with a high specificity.

### 3.8. Assay of CEA in Serum

To prove the applicability of the immunoassay, three standard CEA samples were spiked in 10% fetal bovine serum for analysis. The levels of CEA in the serum samples were determined according to the established standard curve. The found concentrations were close to the added values with acceptable recoveries varying in the range of 96~110% (Table 2). The result suggested that the immunoassay method possesses great potential for the detection of CEA in a complicated matrix.

## 4. Conclusions

Cys-induced aggregation of AuNPs can enlarge the plasmonic signal for target detection. In this work, we developed a plasmonic platform for colorimetric immunoassay of CEA by using PQQ-loaded Cu-BTC MOF to catalyze the oxidation of the aggregation inducer Cys. The dual catalytic reactions promoted by Cu^2+^ and PQQ have remarkably enhanced the sensitivity of immunoassays and allowed for a straightforward readout with the naked eye. The target at a concentration down to 0.01 ng/mL can be readily determined, and the value is comparable to that achieved by other nanomaterials and/or enzymes-based chromogenic reactions for signal readout. Thus, the dual-catalysis system provided a promising means for the design of novel plasmonic biosensors. In addition, we suggested that the target can be anchored on the nanomaterial surface through the metal coordination interaction between the His_6_ tag in protein and the unsaturated Cu^2+^ in MOF. This method should be useful in a myriad of applications ranging from surface modification of nanolabels for point-of-care biomedical diagnostics since many commercially available proteins are recombinant with His_6_ tags.

## Figures and Tables

**Table 1:** Performances of different methods for colorimetric immunoassays of CEA.

Signal Label	Indicator	Linear Range (ng/mL)	Detection Limit (pg/mL)	Ref.
HRP	TMB	0.1−20	30	[60]
NanoHRP	TMB	0.005−1	5	[61]
HRP@PS	TMB	0.1−100	14.347	[62]
HRP-AuNPs	TMB	0.05−50	48	[63]
GOx@ZnCPs	TMB	2−180	50	[64]
Ag_3_PO_4_/Ag	TMB	0.2−2.5	30	[65]
AuNPs/MOF	TMB	0.001−1000	0.742	[66]
TDN-MOF	TMB	0.01–25	3.3	[67]
MoS_2_-AuNPs	4-NP	0.005−10	0.5	[68]
Cu-NFs	PhOH/4-AAP	0.05–40	3.52	[69]
HRP	TMB/AuNRs	Not reported	2500	[70]
cC_3_N_4_/PP	PP	0.5−100	0.34	[71]
Fe_3_O_4_ NPs	Fe^2+^–BPT	0.0001−1000	3.6	[72]
ALP/ZnCPs	Fe^2+^–Phen	0−100	21.1	[73]
Fe_2_O_3_ NPs	Fe^3+^–AAP	0.02–10	11	[74]
g-C_3_N_4_@Fe_3_O_4_	Fe^3+^–salicylate	0.001–50	0.35	[75]
CuO NPs	Cu^2+^–DPHE	0.05−100	26L	[76]
CuO-DNA	HCdS@Au	0.015−2.4	3.5	[77]
Cu-BTC@PQQ	AuNPs	0.01–1	0.01	this work

Abbreviation: HRP, horseradish peroxidase; TMB, 3, 3′,5,5′-tetramethylbenzidine; NanoHRP, HRP nanoparticle; PS, polystyrene microspheres; AuNPs, gold nanoparticles; GOx@ZnCPs, glucose oxidase-integrated zinc(II)-based coordination polymers; TDN-MOF, DNA tetrahedral nanostructures-modified PCN-222 (Fe)-based MOF colloid nanorods; 4-NP, 4-nitrophenol; Cu-NFs, chicken eggwhite-copper phosphate organic–inorganic hybrid nanoflowers; PhOH, phenol; 4-AAP, 4-aminoantipyrine; AuNRs, gold nanorods; PP, phenolphthalein; BPT, bathophenanthrolinedisulfonic acid; Phen, 1,10-phenanthroline; AAP, ascorbic acid 2-phosphate; DPHE, 1,2-diphenyl-2-(2-(pyridin-2-yl)hydrazono)ethenone; HCdS@Au, hollow CdS@Au nanosphere.

**Table 2:** Results for the immunoassays of CEA spiked in serum.

Added (ng/mL)	Found (ng/mL)	**Recovery (%)**
0.01	0.01	100
0.10	0.11	110
0.50	0.48	96

## Data Availability

Data are contained within the article.
